# An IoT Machine Learning-Based Mobile Sensors Unit for Visually Impaired People

**DOI:** 10.3390/s22145202

**Published:** 2022-07-12

**Authors:** Salam Dhou, Ahmad Alnabulsi, A. R. Al-Ali, Mariam Arshi, Fatima Darwish, Sara Almaazmi, Reem Alameeri

**Affiliations:** Department of Computer Science and Engineering, American University of Sharjah, Sharjah P.O. Box 26666, United Arab Emirates; aalnabulsi@aus.edu (A.A.); aali@aus.edu (A.R.A.-A.); g00071630@aus.edu (M.A.); g00069220@aus.edu (F.D.); g00071368@aus.edu (S.A.); g00066638@aus.edu (R.A.)

**Keywords:** visually impaired people, walking assistants, machine learning, IoT, sensors, smartphone

## Abstract

Visually impaired people face many challenges that limit their ability to perform daily tasks and interact with the surrounding world. Navigating around places is one of the biggest challenges that face visually impaired people, especially those with complete loss of vision. As the Internet of Things (IoT) concept starts to play a major role in smart cities applications, visually impaired people can be one of the benefitted clients. In this paper, we propose a smart IoT-based mobile sensors unit that can be attached to an off-the-shelf cane, hereafter a smart cane, to facilitate independent movement for visually impaired people. The proposed mobile sensors unit consists of a six-axis accelerometer/gyro, ultrasonic sensors, GPS sensor, cameras, a digital motion processor and a single credit-card-sized single-board microcomputer. The unit is used to collect information about the cane user and the surrounding obstacles while on the move. An embedded machine learning algorithm is developed and stored in the microcomputer memory to identify the detected obstacles and alarm the user about their nature. In addition, in case of emergencies such as a cane fall, the unit alerts the cane user and their guardian. Moreover, a mobile application is developed to be used by the guardian to track the cane user via Google Maps using a mobile handset to ensure safety. To validate the system, a prototype was developed and tested.

## 1. Introduction

Reports show that at least 2.2 billion people globally have a near or distance vision impairment [1]. A survey-based study on eye disease from January 1980 to October 2018 estimated that in 2020, 43.3 million people were blind, 295 million people had moderate and severe vision impairment, 258 million had mild vision impairment, and 510 million had visual impairment from uncorrected presbyopia [2]. Visual impairment introduces many complications to the affected individuals and their families. Those complications, in turn, directly affect their safety, wellbeing, and independence as well as social responsibilities. A recent study shows that while on the move, 15% of visually impaired people encounter collisions with obstacles such as doors and walls, and 40% may face high fall risk while using stairs, going over hollow pits or up/down hills [3]. The daily life of individuals with visual impairment became even more complex soon after the declaration of the global pandemic due to COVID-19, not only because those individuals are more prone to being infected [4] but also due to the restrictive measures that were globally adopted to mitigate the outbreak of the pandemic [4,5,6]. Several recent studies were conducted to understand the changes in the daily life of a visually impaired individual emerging from the COVID-19 pandemic [4,5,6]. Those studies showed that preventive measures such as mobility restrictions, lockdowns, physical and social distancing, travel restrictions, as well as the use of barrier shield frames in public places have all contributed to major changes in the lives of visually impaired individuals and have also disturbed their support systems. Such disturbances have forced many to individually deal with the newly imposed challenges, especially if they were separated from their main care givers [4,5,6]. One of these studies concluded that using technological tools is a key element in ensuring the continuity of educational and work activities for such individuals [4]. Fortunately, technology has been evolving to cater to visually impaired individuals in terms of navigation and safety as they perform their daily activities independently.

Different types of walking assistants are being used by visually impaired individuals to enable them to move around and perform their day-to-day activities. A recent systematic review was performed to analyze the navigation assistant systems used for blind and visually impaired people [7]. In this review, studies proposing navigation assistants were classified according to the different design approaches used, technology/tools adopted to implement these systems, the mechanisms/applications followed and the parameters considered to ensure the applicability and the reliability of these systems. Another recent survey paper divided the visually impaired walking assistants into three categories, namely sensor-based, computer vision–based, and smartphone-based systems [8]. Moreover, the survey classified the processing power for each system as microcontrollers, microcomputers, or mobile phone processors. The notifications produced by these systems can come in different forms, such as vibrations, beeps, and/or audio. Table 1 shows a summary of the different types of walking assistants according to the technology implemented, sensing/capturing devices utilized, and the computing devices used for processing [8]. Moreover, the table lists the advantages and the disadvantages for each type.

Moreover, many cane products that make use of technology exist in the market. One of the best-selling smart cane products is the Saarthi SmartCane device [9]. The product provides four patterns of vibrations to inform the user of obstacles existing at a distance. The cane allows obstacle detection on ground, surfaces, and potholes. It also provides a battery alert system to warn the user in case the cane is running out of battery. However, this product has several drawbacks. Primarily, although many may view vibrations as a reliable method of relaying information to the blind individual, this feature may cause unwarranted panic to the visually impaired individual. Furthermore, the product cannot detect stairs and doors. Another commercial product that has been developed for blind people and is available in the market is WeWalk [10]. It uses ultrasonic sensors to alert the user by vibrating the cane if there is an obstacle nearby. Furthermore, it navigates using Google Maps, guiding the user to the desired locations through a speaker. Nonetheless, the product does not classify the obstacles encountered. Both commercial products are not suitable for children or the elderly since the guardian cannot track the location of the user.

In this proposed work, a smart cane system was developed to help visually impaired individuals move around independently, easily, and safely. The proposed system has the potential to overcome the limitations of the existing systems by combining the advantages of IoT technology, the emerging machine learning techniques, and mobile applications to provide an optimum service to the users. Thus, the main contributions of this system are the following:

1. Detects and identifies obstacles by integrating a machine learning model within the framework.
2. Generates timely alerts for the cane user and guardian.
3. Provides the guardian with access to a mobile application to keep them aware of the position and status of the visually impaired user for a safe navigation.

The rest of this paper is organized as follows. Section 2 presents the related work. Section 3 describes the methodology, including the hardware and software architecture of the system. In Section 4, the implementation and testing of the systems as well as a comparison with other existing works are discussed. Section 5 concludes the paper. 

## 2. Related Work

Different types of walking assistants have been proposed in the literature to help the visually impaired individuals navigate around and perform their day-to-day activities. Several sensor-based systems have been proposed. In one work, a sensor-based system was proposed to aid the walking of visually impaired people [11]. The system uses an electromagnetic sensor (microwave radar) that was designed and mounted on a traditional white cane to help avoid obstacles. The system was able to detect different obstacles within a range of 1.5 m from the cane. Ramadhan developed a wrist-wearable multiple-sensor system to aid visually impaired and blind people in their navigation [12]. The system utilized multiple components, including a microcontroller, several sensors, cellular communication GPS module, and a self-powered solar panel. In an emergency case, vibration, buzzer sound, and location alarm are sent to the guardian through their mobile phones. In another recent work, a method for detecting low-lying obstacles was proposed [13]. In that work, an assistive cane was used with ultrasonic sensors (US) attached to it and facing the forward direction. Simulations to generate obstacle data and field experiments were all conducted to validate the method. Both the simulation and field experimental results were found to be in agreement. However, this study focused only on obstacle detection and assumed that the cane is firmly held by the visually impaired person and did not take into consideration that the individual may fall during navigation. In another work, a multi-sensor obstacle detection system for a smart cane was proposed to reduce the false alerts delivered to the user [14]. The system consists of five sensors (one accelerometer, three US sensors, and one Infrared sensor (IR)). The sensors are used to measure the distance to the objects, sense the tilt angle of the user, and detect the uneven ground surfaces such as holes and descending stairs. The sensors are attached to a microcontroller in addition to other hardware to deliver alerts (vibration motor and Bluetooth module for wireless audio feedback). The method uses a model-based state-feedback control strategy to regulate the detection angle of the sensors. Real-time experiments were conducted to assess the performance of the system and showed an improvement in terms of error reductions. In another study, a smart path guidance system was developed and tested to guide blind and visually impaired people to walk freely in an unfamiliar environment [15]. The system consists of a simple handheld mobility device to detect objects and their distances from the user. The system uses two US sensors that are placed at 0 and 40 degrees to cover a wider range of area in front of the user. A fuzzy logic design was used on a micro-processor to process the captured information and deliver decisions and warnings. A novel electronic device to help visually impaired people with navigation was proposed [16]. This system is composed of six US sensors, a wet floor sensor, a step-down sensor, microcontroller circuits, four vibration motors and a battery for power supply. All these components are placed on shoes wearable by the user. The system generates a logical map of the surrounding environment, detects obstacles from floor level to knee level, and provides feedback to the user through audio and vibration. An Internet of Things (IoT)-based smart walker device was proposed to reduce the possibility of falling among the elderly and visually impaired people [17]. The system receives information such as the position, objects, and people around the user using sensors, processes this information and sends commands to the controller to guide the user accordingly. Despite their efficiency, sensor-based systems identify a wide range of obstacles, including those that the user may not want to avoid. For instance, the US sensor may consider a staircase as an obstacle that one would steer clear of, while this is not the case. 

Computer vision is a field of computer science that deals with how computers can gain high-level understanding from digital images or videos. Efficient computer vision techniques can run on single board computers, which enables them to be used in several applications such as traffic sign recognition [18], video surveillance [19], obstacle recognition [20], smart waste management [21], mechanical damage identification and classification [22] and energy saving [23]. Several computer vision–based systems have been proposed in the literature to assist visually impaired individuals in their navigation. An embedded computer vision system in a microcontroller platform targeting a wearable application for the visually impaired was proposed [24]. The system was designed to be used as a crosswalk detector that captures and classifies images of crosswalks into four classes (crosswalk on the right, crosswalk on the left, crosswalk straight ahead, and no crosswalk). The method involves capturing images, segmenting Regions of Interest (ROIs), and performing feature extraction using Gray Level Co-occurrence Matrix (GLCM). A Support Vector Machine (SVM) classifier with Radial Basis Function (RBF) kernel was used for classification. Another machine vision guidance system that allows visually impaired individuals to navigate their surroundings instantaneously and intuitively was proposed [25]. The system extracts visual cues from the surrounding environment using a camera and converts them into binaural acoustic cues for users to create cognitive maps. This system uses neural networks and computer vision algorithms to comprehend scenes through object detection, localization and classification. Another study proposed a system that combines both computer vision and mobile application technology to assist visually impaired people in moving around [26]. In this work, an Android application was developed that uses the mobile phone camera of the visually impaired user to detect objects around him/her. The application can detect objects using the object detection API of TensorFlow. Moreover, the application uses computer vision to determine the direction of the object and its distance from the user. It communicates this information to the user via an audio device, such as headphones or speakers. In another work, a 3D object recognition method was implemented on a robotic navigation aid for the detection of indoor structural objects in real time [27]. The system consists of a forward-looking 3D time-of-flight camera and a user-worn mobile computer mounted on a white cane to process the camera data. The object recognition algorithm involves training a Gaussian Mixture Model–based Plane Classifier (GMM) on features extracted from 3D point cloud data. The proposed GMM-based method detects many target objects correctly, with an overall average accuracy of 95.4%, with stairways being classified with 93.3% accuracy and with doorways, a 90.0% accuracy. 

Artificial intelligence (AI) and machine learning methods have been also used to develop systems to aid visually impaired people. For example, a deep learning–based system for real-time surrounding identification was reported [28]. A convolutional neural network (CNN) model was used to identify street signs of public places such as restrooms, pharmacies and metro stations. A dataset of 100 images was collected from each class and used to train the model to identify the surroundings in real time. The accuracy of the proposed method was 90.99%. However, the dataset size used for training was limited, which may affect the accuracy of prediction in other real-life scenarios. Another deep learning–based obstacle detection and classification method was proposed [29]. The model consists of a camera with a pointer attached to it to project a patterned green light grid in the field of view of the camera. The algorithm detects and classifies the obstacles by analyzing the changes in the points’ frame-by-frame movement. The method employed a CNN and a Long-Term Short-Term Memory (LSTM) approach for obstacle detection and classification. The method was tested on six object classes (ground, backpack, box, car, stairs, and concave). The overall accuracy for the multi-class classification was 90.56% (six classes) and 93.87% (four classes) using the frame/clip-based approach and 92.62% (six classes) and 95.97% (four classes) using the majority-based approach, with stairs being classified with 95% accuracy using the frame-based approach and 100% using the majority based approach, considering four classes classification for both approaches. M.W. Rahman et al. presented an IoT-based blind stick system to guide visually impaired individuals [30]. The IoT blind stick functions using a microcontroller that has three US sensors to detect incoming collisions, an accelerator to detect sudden changes in motion, and a camera to capture surrounding obstacle images to inform the caretaker over the cloud. A deep learning technique is used to classify the captured images and inform the user through an audio output. The aforementioned system is divided into two parts each, with a separate microcontroller, which increases its cost and complexity. Another AI-based visual aid system for the completely blind was proposed [31]. The system consists of a camera and sensors for obstacle avoidance, image processing algorithms for object detection, and an integrated reading assistant to read images and produce output in the form of audio. A deep learning approach was adopted for object detection and reading assistance. The system was able to detect multiple objects with an accuracy above 80%. Despite its usefulness, the proposed system lacks advanced features, such as the detection of ascending staircases, the use of GPS or a mobile communication module.

Table 2 summarizes the studies cited in the literature review. The table lists the technology used in each of the studies, the device used, the features provided and the obstacle identification method utilized. As can be seen in Table 2, the proposed method uses both sensor-based and computer vision–based technologies utilizing a single computing unit. Because of this, it is able to detect and identify obstacles, send sound alerts and detect falls. Moreover, it offers remote monitoring by guardians. 

Thus, the proposed system combines the IoT technology and the emerging machine learning techniques to provide smart cane functionality that facilitates independent movement of the visually impaired navigators. It also incorporates mobile applications to provide extra protection to the visually impaired users as well as to allow monitoring by guardians. The proposed system is designed to accomplish the following main functions:

- Detect any obstacle within a predefined distance.
- Classify the obstacles using machine learning, as some obstacles the users may not want to avoid, such as stairs and doors.
- Incorporate a mobile application to allow real-time tracking of the cane user while on the move and alert the user and the guardian in case the cane falls or when help is needed.

In addition to these provided capabilities, the system is designed to be simple, lightweight, function in real-time, and take into consideration the power consumption, which was one of recommended factors in one of the most recent survey papers in the field [7].

## 3. Methodology

The proposed system consists of hardware and software components. Section 3.1 describes the hardware architecture of the system, while Section 3.2 describes the software architecture.

### 3.1. Hardware Architecture of the Proposed System

**Figure 1:**
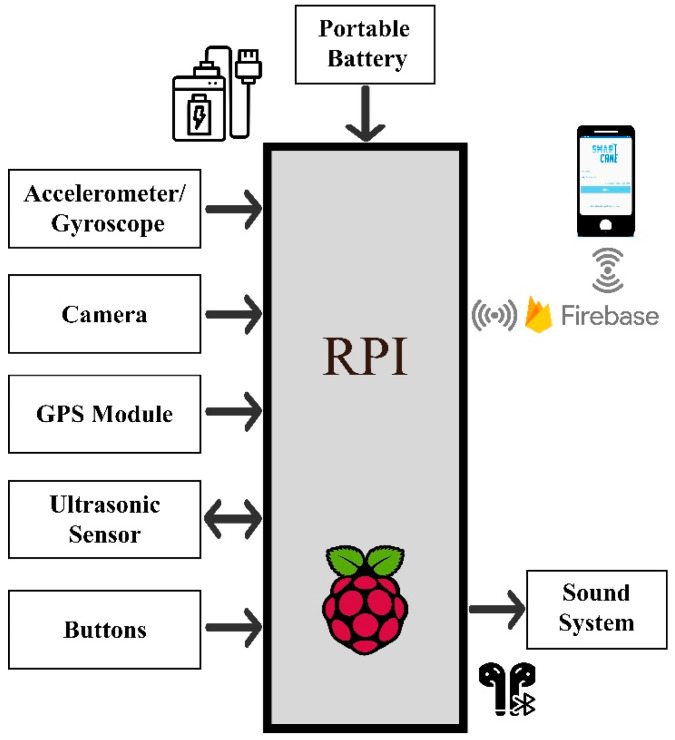
System hardware architecture.

The hardware architecture of the system consists of three main units, namely the sensing and navigation unit, the edge computing unit, and the mobile unit. Figure 1 shows the hardware block diagram of the proposed system. As can be seen in Figure 1, all sensors are connected to the edge computing unit where the computation is performed. Section 3.1.1, Section 3.1.2 and Section 3.1.3 describe the specifications and functionality of the three hardware units used in the proposed system.

#### 3.1.1. Edge Computing Unit

The edge computing unit used in this work is Raspberry Pi (RPi) 4. It is a low-cost computing device of credit card size. It has 64 bits-1.4 GHz CPU, GPU, 4 GB RAM, 32 GB SD memory card, 4 USB Ports, Ethernet, Wi-Fi access points, Bluetooth ports, SPI, SCI, and I2C communication Interfaces.

#### 3.1.2. Sensing and Navigation Unit

The sensing unit consists of a set of sensors, including an accelerometer/gyroscope, cameras, GPS module, US sensors, push buttons and an output sound alarm. The specifications and functionality of each of the sensors are provided as follows:

- Accelerometer/gyroscope (MPU6050): This is used to measure the acceleration and rotation in 3D space. Furthermore, it has an onboard digital motion processor. It can be interfaced to the edge computing device using SPI and I2C communication protocols; the latter was used in the system. The acceleration and rotation sensitivities and resolution are programmable and can be set from 0 to 32,750 units. According to the datasheet, to convert this in terms of degree/sec and g force, the sensitivities are shown in Table 3. Each of these sensors can have four different sensitivities and can be programmed using the FSYNC register. In this work, the most sensitive angular velocity range is 250°/s, and this leads to 131 units/degree resolution. As for the accelerometer, the most sensitive acceleration range is 2 g, and this leads to 16,384 units/g resolution [32].
- Cameras: Two high-quality 8-megapixel resolution cameras are used to capture the surrounding area. One of the cameras is interfaced using the built-in serial camera interface, whereas the other utilizes one of the USB ports of the edge computing device.
- GPS Module: The Vk-162 G-Mouse GPS device is used to track the location of the user. It is interfaced with the edge computing device via one of the USB ports of the edge computing device.
- Ultrasonic (US) Sensors: These sensors are HC-SR04, and they are used to measure the distance between the cane and the surrounding obstacles with a range of 2 cm to 400 cm and with an accuracy of 3 mm. The trigger and echo pins of the sensor are interfaced with the GPIO pins of the edge computing device.
- Sound System: A notification alarm is generated to inform the cane user of the obstacles ahead.
- Push buttons: Three push buttons are used. The first is a power button that is used to power the system. The second is an emergency button used to call for an emergency. The third is an “I am Fine” button, which is used to cancel a false fall alarm.

#### 3.1.3. Mobile Unit

The mobile unit used in this system can be a mobile phone or a tablet. It is utilized to give remote access to the guardian to track and monitor the user. It is worth mentioning that a public cloud computing platform is used to access the cane activities through the guardian mobile set. Table 3 summarizes the specifications of the sensors used in the proposed system.

### 3.2. Software Architecture of the Proposed System

**Figure 2:**
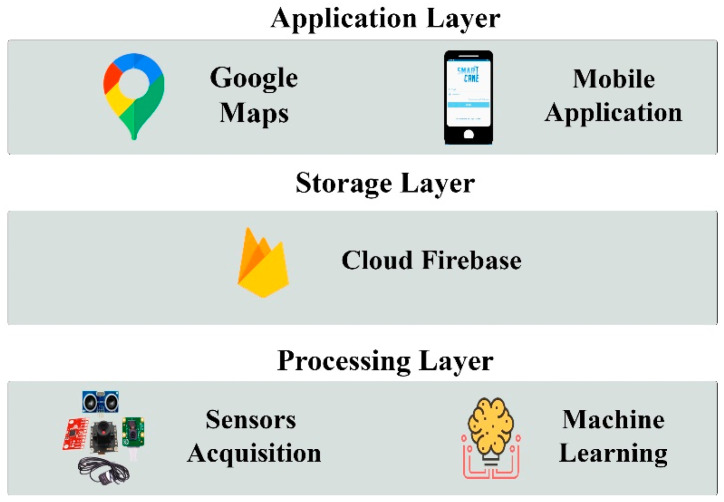
Layered software architecture.

The software architecture of the proposed system consists of three layers, namely the data processing layer (DLP), the data storage layer, and the application layer. Figure 2 shows the layered software architecture. Section 3.2.1, Section 3.2.2 and Section 3.2.3 describe the specifications and functionality of the three software layers used in the proposed system.

#### 3.2.1. Data Processing Layer (DPL)

The DPL consists of two sub-layers, one of which is responsible for the data acquisition from the sensors, and the other is responsible for data analytics. Moreover, the DPL is responsible for updating the data storage layer.

- Sensor Data Acquisition Sub-Layer

This sub-layer consists of several functions, which include configuring, initializing, and reading real-time values from the US sensors, the accelerometer/gyro, and the GPS module, as well as capturing the images using the cameras.

- The distance to the obstacles is calculated using the time interval (t) between sending the sensor trigger signal and receiving that signal by the sensor using the following formula: t/58 = 1 cm.
- The data from the accelerometer/gyro are processed and converted into g force units and rotation in degrees/second, respectively.
- The data from the GPS is processed by extracting the latitude, longitude and time from the raw GPS data.
- The images from the cameras are collected and stored on the RPi for processing and analyzing.

- Machine Learning Sub-Layer

This sub-layer is responsible for processing the input, mainly the captured images, for the purpose of obstacle classification. The processing algorithm is developed using machine learning methods and can be divided into the several stages, namely feature extraction, machine learning methods, model training and prediction.

- Datasets:

The dataset used in this work was taken from a publicly available dataset on GitHub [33]. The dataset consists of 1500 images. These images are classified into four classes: doors (750 images), hollow pits (150 images), downward stairs (300 images) and upward stairs (300 images). Other images (around 50) were captured from the local environment, labeled by the authors with the same classes mentioned above (doors, stairs, and hollow pits) and added to the dataset. Moreover, some of these captured images were taken from the outdoor environment.

- Feature Extraction:

Features describing the shape of the objects were extracted. A Histogram of Oriented Gradients (HOG) Feature Descriptor was used for this purpose [34]. The way the HOG feature extractor calculates the magnitude and direction is explained as follows. It first takes a patch of pixels and generates a pixel matrix for it. Then, it takes each pixel value and compares it to the values of the adjacent pixels to produce two new matrices that denote the gradients G_x_ and G_y_ of each pixel. The bigger the change, the higher the magnitude. Pythagoras’ theorem is used to calculate the magnitude of each pixel [35], as follows:

(1) $$M=\sqrt{\left[{\left(G_{x}\right)}^{2}+{\left(G_{y}\right)}^{2}\right]}.$$

To calculate the orientation, the following formula is used:

(2) $$Ф\;=\mathrm{atan}\left(\frac{G_{y}}{G_{x}}\right).$$

The magnitude M and orientation Ф values are stored in the form of a histogram, where the bins represent the orientations, and the magnitude value in each bin corresponds to a contribution value based on how close its orientation is to this bin or another.

This histogram is built for each block of image of size 8 × 8. The feature extraction algorithm used is sensitive to lightening; thus, the algorithm was applied on smaller blocks to reduce the uncertainties introduced by lightening.

- Machine Learning Methods:

Different machine learning algorithms were used for obstacle classification, including Decision Tree, Naïve Bayes, k-Nearest Neighbor, and SVM [36].

- The Decision Tree Classifier is a tree-structured classifier, where internal nodes represent the features of a dataset, branches represent the decision rules and each leaf node represents the outcome. There are many algorithms used in building decision trees, such as Iterative Dichotomiser 3 (ID3), C4.5—a successor of ID3, and Classification And Regression Trees (CART) [37,38]. The ID3-based algorithms build decision trees using a top-down greedy search approach. A greedy algorithm, as the name suggests, always makes the choice that seems to be the best at that moment. Using these algorithms, decision trees are built as follows. The original dataset of samples is used as the root node. At each iteration of the algorithm, it iterates through the unused features and calculates impurity and information gain (IG). It then selects the feature that minimizes the impurity or maximizes the information gain. After the set of samples is split by the selected feature, the algorithm continues to recur on each subset, considering only features never selected before, until the subsets are pure or a stopping criterion is met. Impurity can be measured using entropy criteria, as follows:
  (3) $$\mathrm{Entropy}=-\sum_{i=1}^{n}p\left(c_{i}\right)\log_{2}\left(p\left(c_{i}\right)\right)$$
  where p(c_i_) is the probability/percentage of class c_i_ in a node.

Information gain (IG) can be defined as the reduction in impurity after splitting the dataset based on a chosen feature. Information gain helps to determine the order of features in the nodes of a decision tree. IG is calculated by comparing the impurity of the dataset before and after the split, as follows:

(4) $$\mathrm{IG}=\mathrm{Entropy}\;\left(D\right)-\sum_{j=1}^{K}\mathrm{Entropy}\;\left(j,\;S\right)$$

where D is the dataset before the split, K is the number of subsets generated by the split, and (j, S) is subset j after the split. Gini index is another impurity measure that is used by other algorithms such as CART to measure impurity.

(5) $$\mathrm{Gini}\;\mathrm{Index}=1-\sum_{i=1}^{n}p^{2}\left(c_{i}\right)$$

- Naïve Bayes classifier is a classification model that is based on Bayes’ theorem, with the “naïve” assumption of conditional independence between every pair of features given the value of the class variable. Bayes’ theorem states the following relationship given class variable y and dependent feature vector x_1_ through x_n_ [39]:
  (6) $$P\left({y|x}_{1},\ldots ,x_{n}\right)=\frac{P\left(y\right)\prod_{i=1}^{n}P(x_{i}|y)}{P\left(x_{1},\ldots ,x_{n}\right)}$$

In this work, the feature values are continuous data, and the likelihood of the features is assumed to be Gaussian, as follows:

(7) $$P\left(x_{i}|y\right)=\frac{1}{\sqrt{2{\pi \sigma }_{y}^{2}}}\exp\left(-\frac{{\left(x_{i}-{\;µ}_{y}\right)}^{2}}{2\sigma_{y}^{2}}\right)$$

where ${µ}_{y}$ and $\sigma_{y}$ are the average and the standard deviation of the class variable y.

- k-nearest neighbor (k-NN) is a non-parametric classification method in which a sample is classified by a majority vote of its neighbors, with the sample being assigned to the class most common among its k nearest neighbors (k is a positive integer, typically small) [40]. If k = 1, then the object is simply assigned to the class of that single nearest neighbor. Distance between the samples is measured using appropriate distance measures. Equation (8) denotes the Euclidean distance between two samples p and q:
  (8) $$D\left(p,q\right)=\sqrt{\sum_{n=1}^{N}{\left(p_{n}-q_{n}\right)}^{2}}$$
  where N is the number of features in the dataset. In this work, k = 96 was selected through experimentation because it was found to yield the best performance.
- Support Vector Machine (SVM) is a powerful classification method that builds a model that maps training samples to points in space such that the width of the gap between the different classes is maximized [41]. Using this model, new samples are then predicted to belong to a class based on which side of the gap they fall. A decision hyperplane, which separates the classes, can be defined by an intercept term b and a decision hyperplane normal vector $\vec{w}$, which is perpendicular to the hyperplane. This vector is commonly referred to in the machine learning literature as the weight vector.

For a set of training data points $=\left\{\left(\vec{x_{i}},{\;y}_{i}\right)\right\}$, where each sample is a pair of a point $\vec{x_{i}}$ and a class label $y_{i}$ corresponding to it, and assuming all points $\vec{x}$ on the hyperplane satisfy ${\vec{w}}^{T}\vec{x}=-b$, the linear classifier for a binary classification problem is

(9) $$f\left(\vec{x}\right)=\mathrm{sign}\;\left({\vec{w}}^{T}\vec{x}+b\right)$$

where the sign for the two classes are −1 and +1.

Since the distance of each sample from the hyperplane is $r_{i}={\;y}_{i}\left({\vec{w}}^{T}{\vec{x}}_{i}+b\right)/\left|\vec{w}\right|$ and the geometric margin is $=\frac{2}{\left|\vec{w}\right|}$, the algorithm aims to find $\vec{w}$ and b such that the geometric margin is maximized (or $\frac{\left|\vec{w}\right|}{2}$ is minimized). The standard formulation of SVM as a minimization problem is defined as follows [41]:

find $\vec{w}$ and b such that

$\frac{1}{2}\;{\vec{w}}^{T}\vec{w}$ is minimized, and

(10) $$\mathrm{for}\;\mathrm{all}\;\left\{\left(\vec{x_{i}},{\;y}_{i}\right)\right\},{\;y}_{i}\left({\vec{w}}^{T}{\vec{x}}_{i}+b\right)\geq 1$$

In addition to performing linear classification, SVMs can efficiently perform non-linear classification using what is called the kernel trick, which is based on implicitly mapping input features into high-dimensional feature spaces. 

**Figure 3:**
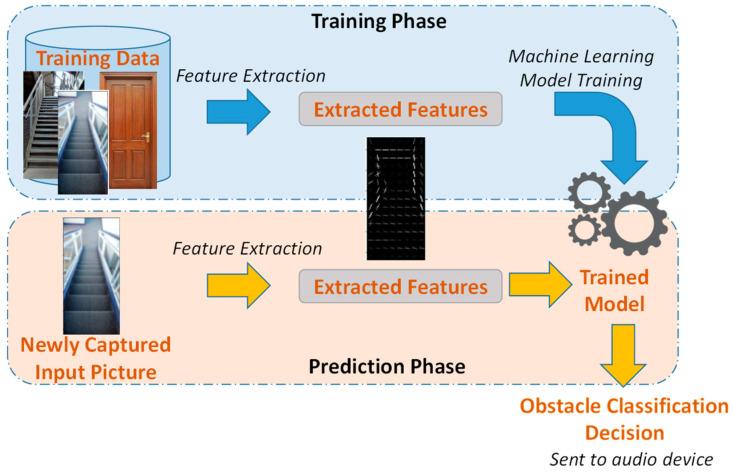
Proposed obstacle classification algorithm.

All the aforementioned algorithms were trained and tested. The experimental results are discussed in Section 4. The best models were chosen to be considered for obstacle classification in real time when obstacles are detected. The images captured by the smart cane are classified, and the cane user is notified of the result in audio. Figure 3 shows the training and testing phases of the obstacle classification algorithm. Since IoT has seen rapid development over the last decade, advanced technologies for computing the scope of objects [42] can be coupled with the machine learning models for identifying obstacles. 

#### 3.2.2. Data Storage Layer (DSL)

This layer uses Firebase cloud storage to store the user GPS location and the emergency notifications that are received from the RPi processing layer through Wi-Fi connection. The guardian can access the database layer through a mobile handset to track the cane user any time and in case of emergency. The Wi-Fi connection utilizes a user certification to allow the clients to access the data stored in the database securely.

#### 3.2.3. Application Layer (AL)

**Figure 4:**
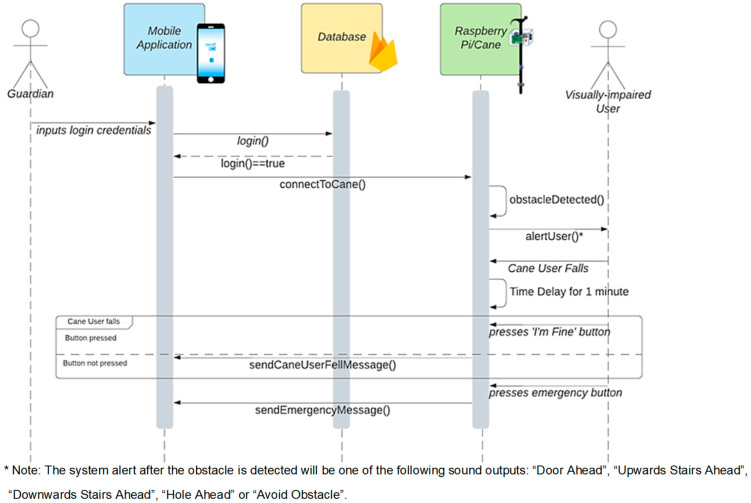
Sequence diagram of the proposed system.

This layer is used to give access to the guardian through his/her mobile handset. The guardian receives the user location and notifications from the cane through the DSL. Using Android Studio, four software modules were developed, namely user interface, user profile and authentication, notification, and GPS tracking using Google Maps. The system sequence diagram is shown in Figure 4 and is described as follows:

- While the visually impaired person is on the move, the sensor data acquisition unit detects obstacles, captures images, and classifies them using the proposed supervised machine learning algorithm. The cane then alerts the visually impaired individual accordingly.
- When a fall is encountered and the “I am Fine” button is not pressed, the cane sends an alarm to the guardian through the cloud database. A notification message is sent to the guardian to act accordingly.
- Once the guardian receives an alert/notification message from the visually impaired individual through the database, he/she can access the database to find out the status and whereabouts of the visually impaired individual via Google Maps.

## 4. System Implementation and Testing

**Figure 5:**
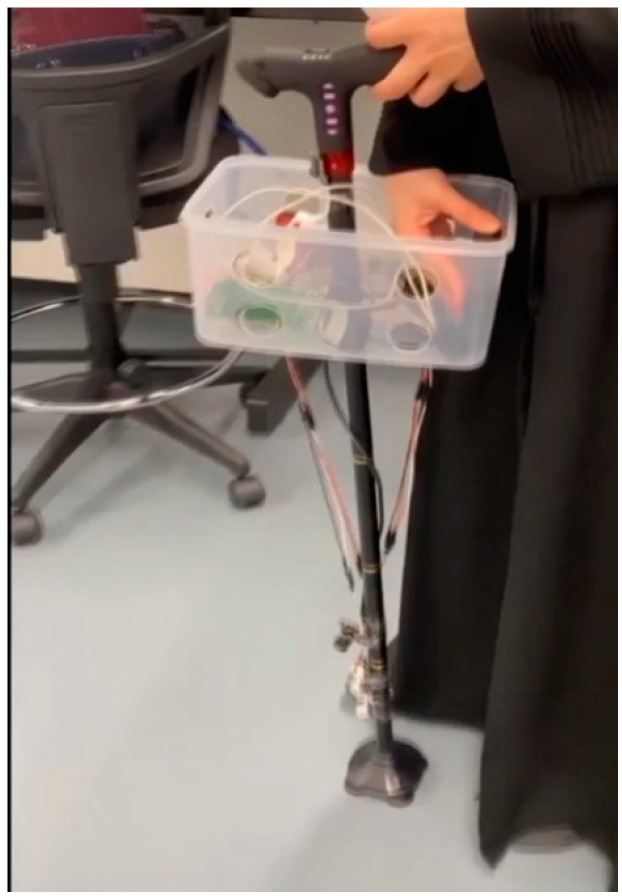
Actual prototype model.

In this section, the implementation and testing details are discussed. To validate the proposed system functionality and performance based on the described hardware and software architectures, a prototype was implemented and tested. The prototype was used by the user. It detects the obstacles, classifies them, notifies the user, reports GPS coordinates, and sends real-time notifications to the guardian in case of emergencies. The prototype was tested with several testing cases that included both indoor and outdoor navigation scenarios. Figure 5 shows the actual prototype model. The system functions were tested and evaluated as follows:

### 4.1. Obstacle Detection

- Distance (Ultrasonic Sensors)

**Figure 6:**
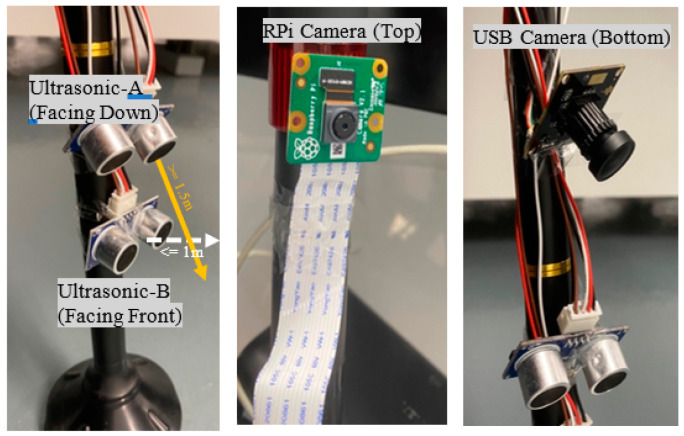
Ultrasonic (US) sensors and cameras.

The smart cane design includes the functionality of obstacle detection. For this purpose, two US sensors are used to detect changes in distances. One is placed perpendicularly, facing the forward direction (US sensor A) to detect obstacles with surfaces approaching as you move towards them, like doors and upward stairs, while the other is oriented towards the ground (US sensor B) to detect obstacles with surfaces that move farther away as you approach them, like hollow pits or downward stairs. Both sensors are mounted at the bottom of the cane. Figure 6 illustrates the placement of the US sensors on the cane.

Specific ranges are given to each US sensor to perform efficiently. These ranges are selected experimentally after several rounds of testing the system. The selected distances are 1 m for US sensor A and 1.5 m for US sensor B. When any of the two US sensors A or B detect a distance less than its specified range, the associated camera is prompted to take a shot. The selected ranges are adjustable and can be changed based on user preferences.

The captured images by the cameras are sent to the machine learning algorithm to identify if the object encountered is a door or an upward stair in the case of Sensor A, or a hollow pit or a downward stair in the case of Sensor B. The user is notified of the type of obstacle in audio format so the user can act accordingly. If the object encountered is not identified by the machine learning algorithm, the cane user will be instructed to avoid the obstacle regardless of its nature for his/her safety.

- Image Capturing (Cameras)

The smart cane design includes two cameras as shown in Figure 6. Each camera is dedicated to a US sensor. The upper camera is associated with the US sensor A, whereas the lower one is associated with US sensor B. When US sensors A or B detect an obstacle, the respective camera takes a shot.

### 4.2. Obstacle Classification

Two classification models were implemented in this work for each of the US sensors and their associated cameras to detect different types of obstacles.

- Feature Extraction Process

**Figure 7:**
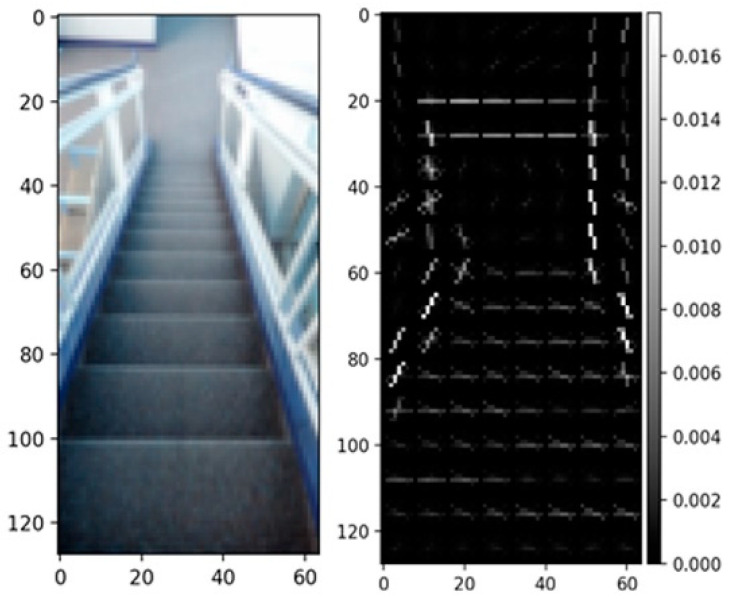
HOG feature descriptor before and after feature extraction.

To extract features from the images for training, the HOG feature descriptor was used. The way this feature descriptor was applied is described as follows:

1. The first step is to resize the images into a smaller size. This is important, as it makes all images equal in size and faster to process. All images are resized to 128 × 64 prior to extraction.
2. To extract HOG, the skimage.feature library is imported [43]. A total of 3780 features were extracted from each image. Figure 7 shows a sample of a resized dataset image and its corresponding HOG representation.

- Model Selection and Training

As mentioned in Section 3, four different classification algorithms were applied and compared. Five-fold cross-validation was used to evaluate the models. The parameters for all the models were tuned using a grid search. The performance metrics results for all the machine learning methods in model 1 and model 2 are shown in Table 4 and Table 5, respectively. The SVM with RBF kernel classifier showed the best performance in both models, with a prediction accuracy of 99% in model 1 and 100% in model 2. 

**Figure 8:**
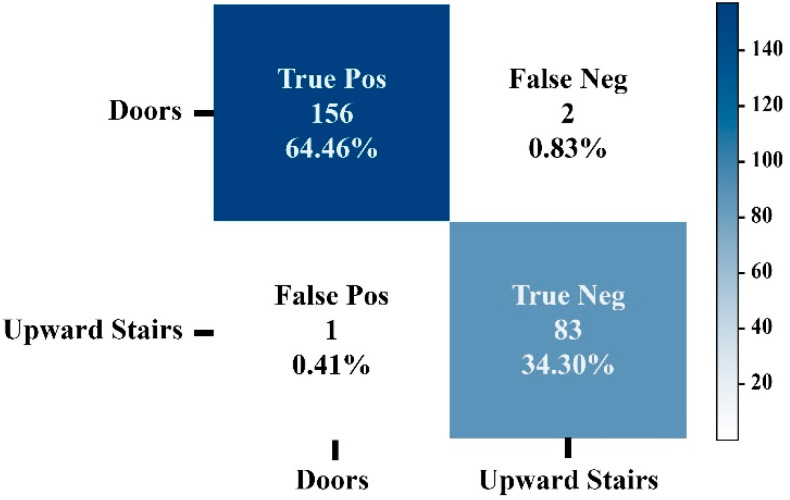
Confusion Matrix of Model 1 using SVM (doors vs. upward stairs).

**Figure 9:**
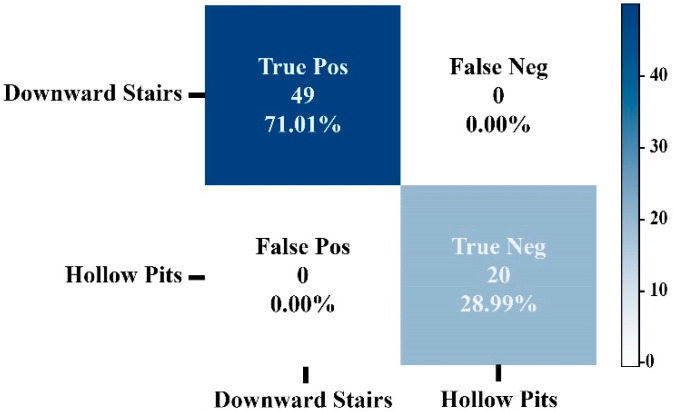
Confusion Matrix of Model 2 using SVM (downward stairs vs. hollow pits).

Figure 8 shows the confusion matrix of model 1 using the SVM classification algorithm. As can be noticed, only three images were classified falsely out of 242 test images. Similar results were obtained for the second model. Figure 9 shows the confusion matrix of model 2. As can be seen from Figure 9, all images were classified correctly. Thus, the trained SVM models were considered and uploaded on the smart cane and used for real-time prediction. 

Thus, the classification accuracy for the proposed system is 99–100% for the obstacles considered in this work (stairs, doors and hollow pits), which was found to be significantly higher than similar works reported in [27,29]. In [27], the average classification accuracy using the GMM classifier was 93.3% for stairways and 90.0% for doorways. In [29], the authors conducted several experiments using different methods. The highest classification accuracy for stairs was 95% using the frame-based approach (similar to the approach adopted in our work). However, they achieved a classification accuracy of 100% for stairs, but using the majority-based approach, whose input is a clip instead of a single frame (the method decides the final classification based on the majority of votes taken from each frame in the entire frame sequence/clip). Unlike our work, the works in [27] and [29] did not consider classifying hollow pits and did not distinguish between upward and downward stairways.

### 4.3. Fall Detection

The smart cane design integrates a fall detection functionality in order to detect when a cane falls and identifies it as an emergency situation. To detect a fall, the six-axis accelerometer/gyro motion-tracking sensor is used. Using this sensor, the retrieved acceleration readings enable the system to detect when the cane falls.

Through trial and error, readings in the *x*, *y*, and *z* axis of the acceleration that imply that the cane had fallen were set. If those values are encountered, a function is triggered to send a notification message to the guardian. However, sending the notification is paused for a duration of one minute to avoid calling the guardian if the fall is not serious, as the cane user has the option to press the “I am Fine” button on the cane to cancel sending the message. If the button is not pressed within one minute, the message will be sent. 

### 4.4. GPS Tracking

GPS tracking is one of the main functionalities required in the design of the proposed system. It is important for the mobile application user (guardian) to know the location of the cane user in case an emergency occurs. Therefore, through integrating the VK-162 G-Mouse USB GPS Module (Beitian Co. Limited, China) into the hardware, the guardian will have real-time updates on the location of the cane user [44]. The GPS module is used to extract the latitude and longitude. 

From the mobile application side, the cane is connected to the mobile app to give the guardian access to the GPS location of the cane user. Hence, each cane is given a unique ID that is stored in Firebase. The guardian can make a connection by typing that ID in a provided field in the mobile app.

**Figure 10:**
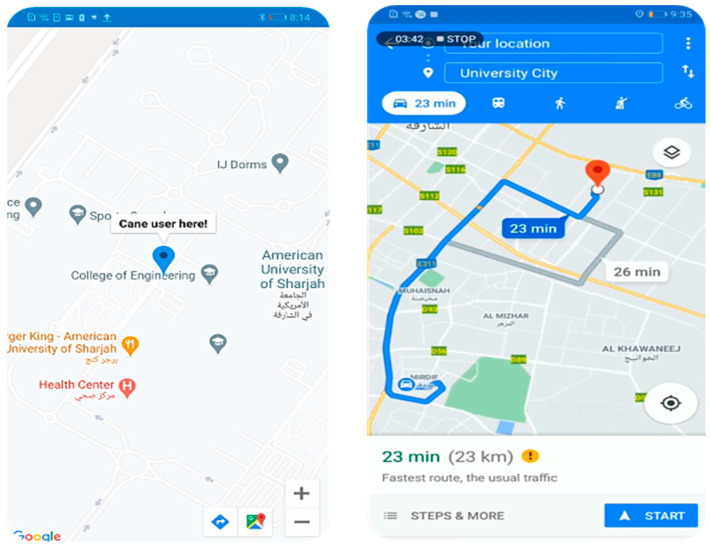
GPS tracking in mobile app.

A Google Maps API key and a Firebase query are used to obtain the latest location shared. Once the tracking option is selected, after connecting the cane, the guardian is directly forwarded to the Google Maps app, where the latest retrieved location is marked. The guardian uses the marker to obtain the directions to the cane user using the Google Maps app. Figure 10 shows samples of GPS tracking and Google Maps used in the app.

### 4.5. Guardian Notification

**Figure 11:**
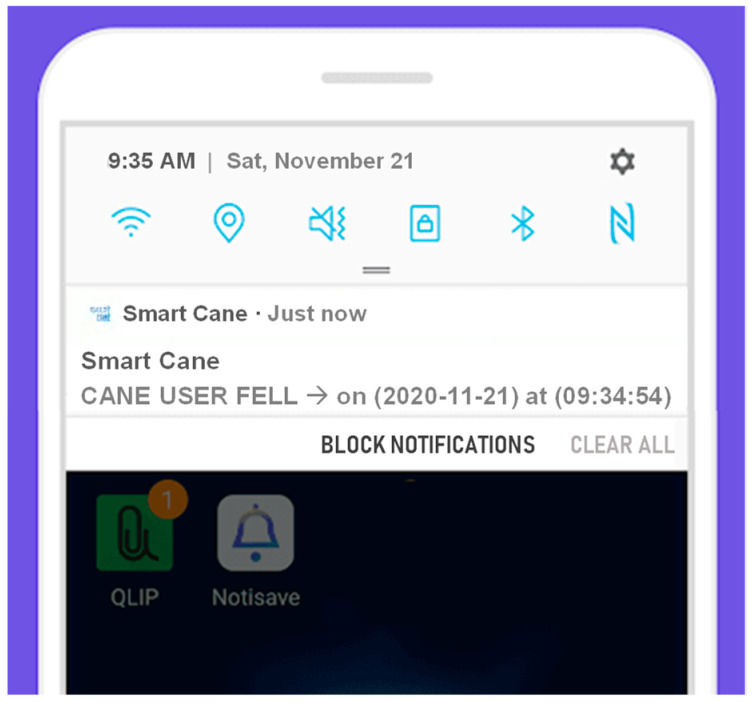
Notifications in the mobile app.

After connecting to the cane, the guardian will be able to receive notifications sent from the cane in real time. The notifications used in this app are foreground notifications. This enables the app user to receive notifications even after closing the app. A broadcast receiver was also utilized to allow notifications to run immediately after restarting the phone. Figure 11 presents a sample of a notification received due to a fall encountered.

## 5. Conclusions

In this work, a smart IoT-based system is proposed to empower visually impaired people and help them navigate around independently and safely. The smart cane consists of a mobile sensor unit that can be attached to an off-the-shelf cane to collect information about the cane user and the surrounding area and deliver alarms to the cane user during navigation and to the guardian in case of emergency. The sensors used include a digital motion sensor, six-axis accelerometer/gyro, US sensors, GPS sensor, and a camera, which are all interfaced with a single-board microcomputer. Machine learning is used by the system to identify obstacles and alarm the user about their nature. Moreover, a mobile application is developed to be used by the guardian to track the cane user via Google Maps using a mobile handset. The single-board Wi-Fi communication between the user and the guardian is established via cloud-based storage that hosts the cane operation and the information of the object surroundings. A prototype of the proposed system was implemented and tested. The obstacle detection module outperformed other modules in the literature.

## Figures and Tables

**Table 1:** Summary of the different types of walking assistant systems.

Technology	Sensing/Capturing Devices	Computing Devices	Advantages	Disadvantages
Sensor-based	US, IR, motion, laser radar sensors, RFID sensors	Microcontroller, System-on-chip	Low cost, fast detection, easy to install, easy to use, suitable for wayfinding	Bulky, US readings may change with temperature and humidity, no identification of obstacles
Computer Vision–based	Depth, vision, RGB cameras, other sensors	Single board computer, laptop, PC	Detects and identifies objects, real-time data acquirement and training, lightweight	High cost, camera works poorly outdoors (because of sunlight), classification works poorly in crowded areas, slow response time due to processing
Mobile Phone–Based	Phone cameras, accelerometers, gyroscopes, magnetometers, GPS proximity sensors	Mobile phone processor	Low cost, efficient, compact, covers indoor and outdoor areas	Not configurable, performs poorly in elevated areas, short detection range

**Table 2:** Summary of visually impaired walking assistance systems.

Ref. No.	Technology	Device	Features					Obstacle Identification
			Obstacles		Alerts	Remote Monitoring and Guardian Alert	Fall Detection	-
			Detect	Identify				
[11]	Sensor Based	Cane	Yes	No	Yes	No	No	-
[12]	Sensor Based	Wearable	Yes	No	Yes	SMS	Yes	-
[13]	Sensor Based	Cane	Yes	No	No	No	No	-
[14]	Sensor Based	Cane	Yes	No	Yes	No	No	-
[15]	Sensor Based	Hand-held	Yes	No	Yes	No	No	-
[16]	Sensor Based	Shoe	Yes	No	Yes	No	No	-
[17]	Sensor Based	Walker	Yes	No	Yes	Mobile App	No	-
[24]	Computer Vision Based	Wearable	No	Yes	No	No	No	GLCM-based crosswalk identification
[25]	Computer Vision + Mobile Phone Based	Smartphone	Yes	Yes	Yes	No	No	Neural network–based object identification
[26]	Computer Vision + Mobile Phone Based	Smartphone	Yes	Yes	No	No	No	TensorFlow-based object identification
[27]	Computer Vision Based	Cane	Yes	Yes	Yes	No	No	GMM-based object identification
[28]	Computer Vision Based	Hand-held	No	Yes	No	No	No	CNN-based street sign identification
[29]	Computer Vision Based	Hand-held	Yes	Yes	No	No	No	CNN- and LSTM-based object identification
[30]	Sensor Based + Computer Vision Based	Cane and Cap	Yes	Yes	Yes	Mobile App	Yes	Mask R-CNN–based object identification
[31]	Sensor Based + Computer Vision Based	Eyeglasses	Yes	Yes	Yes	No	No	TensorFlow-based object identification
**Proposed system**	**Sensor Based + Computer Vision Based**	**Cane**	**Yes**	**Yes**	**Yes**	**Mobile App**	**Yes**	**SVM-based object identification**

**Table 3:** The specifications of the sensors used in the proposed system.

Device Name	Full Scale Range	LSB Sensitivity
Accelerometer 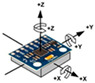	±2 g, ±4 g, ±8 g, ±16 g	16,384 LSB/g, 8192 LSB/g, 4096 LSB/g, 2048 LSB/g
Gyroscope 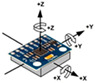	±2500/s, ±5000/s, ±10,000/s, ±20,000/s	131 LSB/°/s, 65.5 LSB/°/s, 32.8 LSB/°/s, 16.4 LSB/°/s
Ultrasonic Sensor 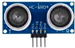	2 cm to 400 cm	3 mm
Camera 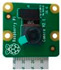	8 megapixel	1080p × 30
GPS 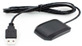	2 m	1–10 times/s (refresh rate)

**Table 4:** Performance metrics for machine learning algorithms used in model 1.

Classification Algorithm		Decision Tree	Naïve Bayes	SVM	K-Nearest Neighbor
Performance Metrics	Accuracy	0.89	0.93	0.99	0.98
	Recall	0.91	0.93	0.99	0.98
	Precision	0.91	0.94	0.99	0.98
	F-Score	0.91	0.93	0.99	0.98

**Table 5:** Performance metrics for machine learning algorithms used in model 2.

Classification Algorithm		Naïve Bayes	SVM	K-Nearest Neighbor
Performance Metrics	Accuracy	0.83	1.00	0.83
	Recall	0.83	1.00	0.83
	Precision	0.88	1.00	0.88
	F-Score	0.83	1.00	0.83

## Data Availability

Not applicable.
